# Published Reports of Delayed Hemolytic Anemia After Treatment with Artesunate for Severe Malaria — Worldwide, 2010–2012

**Published:** 2013-01-11

**Authors:** Melissa Briggs, Paul M. Arguin

**Affiliations:** Div of Parasitic Diseases and Malaria, Center for Global Health, CDC

Artesunate has been recommended by the World Health Organization (WHO) as the first-line treatment for severe malaria since 2010. It is not licensed in the United States but is available from CDC under an investigational new drug (IND) protocol. During 2010–2012, a total of 19 cases of delayed hemolytic anemia after treatment of severe malaria with artesunate were published in the peer-reviewed medical literature, but no such cases have been reported in the United States. CDC Malaria Branch staff reviewed each published report of delayed hemolysis after artesunate use. Based on the pathogenesis of malaria, the hemolysis likely is a result of severe malaria and not the treatment itself. However, artesunate used in the United States is produced by the U.S. Army Medical Materiel Development Activity, and artesunate used outside of the United States is not. An unrecognized difference might exist between the U.S. artesunate and the artesunate used elsewhere. Alternatively, cases of artesunate-associated hemolysis might have occurred in the United States but were not reported. To better assess these possibilities, CDC has amended the artesunate IND protocol and now recommends that persons treated for severe malaria with artesunate be followed for 4 weeks after treatment and evaluated for hemolytic anemia.

A literature search was performed using the terms “artesunate” and either “hemolytic anemia” or “delayed anemia.” All reports were reviewed, and additional case reports referenced in these reports also were obtained and reviewed. Case reports were considered relevant if the patient had received artesunate therapy for the treatment of severe malaria and then experienced worsening hemolytic anemia after initial clinical improvement and resolution of parasitemia. In total, six articles (describing 19 cases) fit these requirements. One incident of hemolytic anemia was reported in two different articles, one published in 2002 and the other in 2010. Only the 2010 report is included in this summary.

All cases except one were in adults (median age: 50 years; age range: 5–71 years) (Table). Eighteen patients had traveled to sub-Saharan Africa, and one to India. All 12 patients with documentation of parasitemia were hyperparasitemic (>5% of red blood cells [RBCs] infected), with a mean highest parasitemia reported of 22% (range: 7%–45%). Seven of the patients also received artemether-lumefantrine, an oral drug similar to artesunate, for either initial treatment or to complete the treatment regimen. Ten patients received nonartemisinin antimalarials before or after artesunate therapy. All 14 patients for whom multiple hemoglobin measurements were available had a reduction in hemoglobin within the first week of treatment, from a mean of 12.3 g/dL on admission to 8.8 g/dL on follow-up. Seven patients received transfusions with either packed RBCs or platelets during their initial presentation, including three who received exchange transfusions. All patients responded well to artesunate therapy, and complete parasite clearance was documented on average by day 5.

For all patients, hemolysis and worsening anemia were described after parasite clearance, 8–32 days after completion of artesunate therapy. The mean hemoglobin nadir was 6.2 g/dL (range: 4.4–8.6 g/dL) (n = 18). Other reported laboratory measurements supported the diagnosis of hemolysis including elevated lactate dehydrogenase, low or absent haptoglobin, elevated bilirubin, and/or elevated reticulocyte count (n = 12). Twelve patients required transfusions, ranging from 2–24 units, after artesunate treatment. The hemolysis resolved, and hemoglobin improved in all patients within 4–8 weeks after artesunate therapy. Two patients had documented moderate anemia more than 30 days after treatment.

Two studies included information on patients with severe malaria both with and without delayed hemolysis. One reported that the six patients with hemolysis had received a higher mean cumulative dose of artesunate than the 19 patients without hemolysis (12.8 mg/kg versus 7.6 mg/kg) (1). However, the mean cumulative dose in patients with hemolysis in the other article was 7.2 mg/kg, and no correlation between dose and hemolysis was observed (2). Although not specifically discussed, the reported parasitemia in the patients with delayed hemolysis was higher in both studies (mean parasitemia of 16% versus 11%, and median parasitemia of 27% versus 5%, respectively).

## Editorial Note

This review of published cases found reports of 19 cases of delayed hemolysis after artesunate therapy for severe malaria in nonendemic countries outside of the United States. Thus far, no published studies have assessed whether artesunate actually causes or increases the risk for delayed hemolysis; therefore, it remains unknown whether the hemolysis described is a direct effect of the treatment or simply a consequence of severe malaria itself.

In 2010, a total of 176 cases of severe malaria in the United States were reported to CDC, 39 (22%) of which were in patients who received artesunate through the CDC IND protocol (3). Thus far, delayed hemolytic anemia has not been reported in patients treated with artesunate in the United States. Artesunate has been recommended by WHO as first-line treatment for severe malaria since 2010. It has been shown to be superior to quinine, with increased survival and decreased adverse events (4). Recently, many nonendemic countries have begun to use artesunate in patients with travel-associated severe malaria. The United States is the only country where the artesunate used has been certified as meeting good manufacturing practice (GMP) standards. In all other countries, the only form of artesunate available has not been certified as having been produced according to GMP standards, but has been prequalified by WHO as an essential drug (1,5). WHO prequalification involves a review of safety data and a manufacturing site assessment but is not thought to be as stringent as GMP certification. Various authors have expressed concern that these delayed hemolytic events might be a direct toxicity of the non-GMP artesunate that is currently used outside of the United States (1,2). However, based on this review on malaria and hemolysis, there appear to be more compelling mechanisms that might explain these delayed hemolytic events related to the pathogenesis of severe malaria itself.

There have been multiple published reports of hemolytic anemia in malaria not associated with artesunate. These include reports of “blackwater fever” (dark red or black urine associated with acute malaria) and prolonged hemolytic anemia in severe malaria patients treated with older antimalarial medications. In 1979, one study showed that circulating RBCs in patients with acute malaria continued to have a decreased life-span for up to 4–5 weeks after parasite clearance. Patients in this study treated with chloroquine experienced a mean 9% decline in their hemoglobin, occurring 3–22 days after completion of their treatment. Researchers also detected mild suppression of RBC production and complement-containing immune complexes on RBC surfaces after infection, likely promoting increased splenic removal of RBCs (6). A study conducted in The Gambia showed that patients with severe malaria, but without severe anemia, experienced an initial drop in hemoglobin when started on treatment. In addition, in a subset of children without hemoglobinopathies or glucose-6-phosphate dehydrogenase deficiency (n = 17), 16 were direct antiglobulin test (DAT)–positive, and nine were positive for immunoglobulin G autoantibodies, with higher parasitemia being associated with increased DAT positivity (7). In 1993, researchers in Germany detected immunoglobulin M antiglycolytic antibodies in patients with severe malaria and prolonged hemolysis after parasite clearance. These antibodies were only present in patients with severe malaria from *Plasmodium falciparum*, persisted up to >40 days after treatment, and resolved as hemolysis resolved. The typical hemoglobin nadir in this study occurred 6–12 days postdiagnosis (8).

What is already known on this topic?

Recent reports of delayed hemolytic anemia after artesunate treatment for severe malaria in nonendemic countries other than the United States have generated concern that this phenomenon might be related to the treatment.

What is added by this report?

Published reports describing prolonged hemolytic anemia in severe malaria not associated with artesunate treatment suggest multiple possible causes related to the pathogenesis of severe malaria infection itself.

What are the implications for public health practice?

Additional data are needed. Clinicians treating patients for severe malaria with artesunate should monitor the patient for 4 weeks after treatment, assess for hemolysis if anemia is present, and report any episodes of delayed hemolysis to CDC.

In 1997, a study using antibodies against ring-infected erythrocyte surface antigens (RESA) to label *P. falciparum*–infected cells found that RBCs with the RESA antigen, but without parasites, could be detected if labeled in vivo. This led to the hypothesis that the spleen or another organ removed or killed the parasites without destroying the RBCs (9). A follow-up study found that, in patients with severe malaria, artesunate treatment generated a much higher number of unparasitized RESA-positive RBCs than quinine. It also noted that artesunate-treated RBCs were more deformable then quinine-treated or parasitized RBCs, likely further extending their lifespan (10). This increase in RBCs surviving after parasitemia, albeit with a shorter lifespan than healthy RBCs, might explain the delayed postartesunate treatment decrease in hemoglobin observed in the cases reported.

Further comparative data would be required to determine how artesunate influences the risks for this complication, both in travelers and in the increasing proportion of patients receiving artesunate for severe malaria in highly endemic countries. There is insufficient evidence at present to attribute these few reported hemolytic events directly to artesunate treatment itself, and these events should not reduce confidence in artesunate, which has many other benefits for patients with severe malaria. In addition, all patients with reported delayed hemolysis have recovered without long-term complications. However, to further understand the relationship between delayed hemolysis and artesunate use, CDC is requesting that patients treated with artesunate in the United States be evaluated and have their hemoglobin assessed 4 weeks after treatment. Significant declines in hemoglobin should be reported to CDC’s Malaria Branch and should prompt an evaluation for hemolysis and closer monitoring.

## Figures and Tables

**TABLE:** Articles and cases reported in the literature involving delayed hemolytic anemia after treatment with artesunate, by selected characteristics — worldwide, 2010–2012

Case	Location	Case description	Peak parasitemia reported	Initial treatment	Artesunate total dose	Follow-up treatment	Parasite clearance time	Initial Hgb (g/dL)	Hgb nadir (g/dL)	Day of nadir*	Other laboratory findings
**Rolling T, Schmiedel S, Wichmann D, et al. Post-treatment haemolysis in severe imported malaria after intravenous artesunate: case report of three patients with hyperparasitaemia. Malar J 2012;11:169.**											
Case 1	Germany	German female aged 19 yrs with hypotension and hyperparasitemia	14%	Mefloquine	8 mg/kg	Mefloquine	5 days	12.0	8.6	14	LDH 1,010
Case 2	Germany	German male aged 54 yrs with somnolence, hypotension, hyperbilirubinemia, hyperparasitemia, and acute renal failure	21%	Artesunate	9 mg/kg	Atovaquone/proguanil	7 days	NA	5.7	14	LDH increased Haptoglobin absent DAT IgG+
Case 3	Germany	German male aged 55 yrs with fever, acute renal failure, hyperbilirubinemia, and hyperparasitemia	20%	Artesunate	9 mg/kg	Atovaquone/ proguanil	NA (<1% on day 3)	14.7	6.6	15	LDH increased Haptoglobin absent DAT negative
**Kreeftmeijer-Vegter AR, van Genderen PJ, Visser LG. Treatment outcome of intravenous artesunate in patients with severe malaria in the Netherlands and Belgium. Malar J 2012;11:102.** †											
Case 4	Netherlands/ Belgium	Male aged 53 yrs with jaundice, impaired consciousness, and hyperparasitemia	34%	Quinine	NA	Atovaquone/ proguanil	4 days	12.9	6.9	20	DAT C3d+
Case 5	Netherlands/ Belgium	Female aged 50 yrs with impaired consciousness, jaundice, acidosis, renal impairment, and hyperparasitemia	19%	Artesunate	NA	Atovaquone/ proguanil	3 days	14.0	7.0	30	NA
Case 6	Netherlands/ Belgium	Female aged 50 yrs with jaundice and hyperparasitemia	11%	Artesunate	NA	Atovaquone/ proguanil	3 days	11.4	4.5	13	DAT negative
Case 7	Netherlands/ Belgium	Female aged 44 yrs with hyperparasitemia	37%	Quinine	NA	Artemether-lumefantrine	4 days	9.7	6.1	15	DAT C3d+ IgG+
Case 8	Netherlands/ Belgium	Male aged 5 yrs with shock, impaired consciousness, and hyperparasitemia	12%	Quinine	NA	Artemether-lumefantrine	4 days	9.8	6.1	8	DAT negative Hemacult negative
Case 9	Netherlands/ Belgium	Female aged 50 yrs with jaundice, hemoglobinuria, and hyperparasitemia	30%	Artesunate	NA	Artemether-lumefantrine	10 days	11.6	6.9	13	DAT IgG+ IgM+ Hemacult negative G6PD normal
Case 10	Netherlands/ Belgium	Female aged 71 yrs with impaired consciousness, acidosis, hypoglycemia, respiratory distress, renal impairment, and hyperparasitemia	20%	Quinine	NA	Artemether-lumefantrine	7 days	8.1	6.0	13	DAT negative
**Zoller T, Junghanss T, Kapaun A, et al. Intravenous artesunate for severe malaria in travelers, Europe. Emerg Infect Dis 2011;17:771–7.**											
Case 11	Europe	Female aged 30 yrs with hyperparasitemia	20%	Artesunate + doxycycline	12 mg/kg	None	79 hrs	11.3	5.7	15	LDH 1,437 Reticulocytes 10.2% DAT negative G6PD normal
Case 12	Europe	Female aged 54 yrs with HIV, admitted with hyperparasitemia and cerebral malaria	20%	Artesunate + doxycycline	12 mg/kg	None	158 hrs	13.2	6.1	32	LDH 805
Case 13	Heidelberg, Germany	Male aged 32 yrs with hyperparasitemia	30%	Artesunate	12 mg/kg	Atovaquone-proguanil	104 hrs	13.4	5.3	19	LDH 672
Case 14	Helsingborg, Sweden	Male aged 46 yrs with diabetes mellitus admitted with malaria, renal failure, and jaundice	4%	Artesunate	20 mg/kg	None	48 hrs	13.4	7.8	15	LDH 660 Reticulocytes increased
Case 15	Bergen, Norway	Male aged 49 yrs with renal failure, jaundice, disseminated intravascular coagulation, and hyperparasitemia	9%	Artesunate + doxycycline	12 mg/kg	Artemether-lumefantrine	35 hrs	15.5	5.7	15	LDH 1,489 Reticulocytes increased Haptglobin <0.1
Case 16	Europe	Female aged 34 yrs with renal failure, shock, and hyperparasitemia	10%	Artesunate + doxycycline	10 mg/kg	Artemether-lumefantrine	NA	14.2	5.8	16	LDH 444 Reticulocytes increased Haptoglobin <0.8 G6PD normal
**Caramello P, Balbiano R, De Blasi T, et al. Severe malaria, artesunate and haemolysis. J Antimicrob Chemother 2012;67:2053–4.**											
Case 17	Italy	Italian woman aged 22 yrs with fever, splenomegaly, jaundice, and hyperparasitemia	7%	Artemether-lumefantrine	NA	NA	4 days	NA	5.6	13	LDH 2,406 DAT negative G6PD normal
**Kano S. Artemisinin-based combination therapies and their introduction in Japan. J Infect Chemother 2010;16:375–82.**											
Case 18	Japan	Japanese woman aged 68 yrs	45%	Artesunate	NA	NA	1 day	NA	NA	11	NA
Case 19	Japan	Japanese man aged 54 yrs	NA	Artesunate	NA	NA	1 day	NA	4.4	15	LDH 1,483

**Abbreviations:** Hgb = hemoglobin; LDH = lactate dehydrogenase (in U/L); DAT = direct antiglobulin test; G6PD = glucose-6-phosphate dehydrogenase; NA = not available; IgG = immunoglobulin G; IgM = immunoglobulin M.

* Day of nadir = days after first dose of artesunate.

† Initial Hgb, for this article only, interpreted from published graphs of hemoglobin over time.
